# Machine learning identifies molecular targets of Di (2-ethylhexyl) phthalate in pulmonary arterial hypertension

**DOI:** 10.3389/fbinf.2026.1711637

**Published:** 2026-03-20

**Authors:** Hua Li, Yingchun Jiang, Jijia Li

**Affiliations:** 1 Department of Pulmonology, Nanxishan Hospital of Guangxi Zhuang Autonomous Region, Guilin, China; 2 Department of Radiology, Nanxishan Hospital of Guangxi Zhuang Autonomous Region, Guilin, China; 3 Department of Cardiology, Nanxishan Hospital of Guangxi Zhuang Autonomous Region, Guilin, China

**Keywords:** bioinformatics, di(2-ethylhexyl) phthalate, machine learning, molecular targets, pulmonary arterial hypertension

## Abstract

**Objective:**

This study aims to explore the potential molecular mechanisms by which di (2-ethylhexyl) phthalate (DEHP) exposure induces pulmonary arterial hypertension (PAH).

**Methods:**

We conducted differential expression analysis on multiple genomics datasets to pinpoint PAH-associated genes. Subsequently, an integrative approach combining machine learning algorithms and network toxicology was employed to examine the binding interactions between DEHP and the identified target proteins.

**Results:**

Our analysis identified 60 genes as potential targets of DEHP in PAH. Further refinement using machine learning prioritized twelve core regulatory genes: ALKBH2, AOC2,BCL2L10,CTBP2,DNM2,ERLIN2,HPS6,RABGGTA,PON2,SLC4A7,SORT1, and PDE4D. Among these, HPS6, CTBP2,RABGGTA, SORT1,ALKBH2,BCL2L10, AOC2,and PON2 were significantly downregulated, whereas SLC4A7,PDE4D, ERLIN2,and DNM2 were markedly upregulated (P < 0.05).

**Conclusion:**

These findings demonstrate that DEHP promotes PAH pathogenesis by modulating specific genes and associated pathways. The twelve core genes identified through machine learning are proposed as key regulators in this process, providing crucial insights for future mechanistic investigation into DEHP-induced PAH.

## 1 Introduction

PAH is a rare, progressive, and prognostically severe cardiopulmonary disorder characterized by sustained elevation of pulmonary artery pressure, ultimately leading to right heart failure and death (Jutant and Humbert, 2016; Tuder et al., 2013). Its pathophysiology is complex and multifactorial, arising from a dysfunctional interplay between genetic predisposition, aberrant cellular processes in the pulmonary vasculature (involving endothelial dysfunction, smooth muscle cell proliferation, and inflammation), and environmental exposures (Jutant and Humbert, 2016; Tuder et al., 2013; Hou et al., 2019; Zhou et al., 2022). While mutations in genes such as BMPR2 are established heritable risk factors, and conditions like connective tissue diseases are known associated causes, a significant proportion of PAH etiology remains unexplained, underscoring the critical role of environmental modifiers (Mocumbi et al., 2024).

Among environmental risk factors, the ubiquitous plasticizer DEHP has garnered increasing concern. Epidemiological and experimental studies have linked phthalate exposure to adverse vascular outcomes and endothelial impairment (Chu et al., 2021). DEHP, often coexisting with other pollutants like polycyclic aromatic hydrocarbons (PAHs) in particulate matter, is postulated to contribute to pulmonary injury through mechanisms involving oxidative stress and the induction of pro-inflammatory cytokines (e.g., IL-6, IL-8) (Ataei et al., 2022). For instance, co-exposure to DEHP and benzo [a]pyrene has been shown to synergistically enhance mitochondrial reactive oxygen species (ROS) and inflammatory responses in cellular models, suggesting a plausible pathway for pulmonary vascular damage (Hou et al., 2019).

However, a significant knowledge gap persists. Current evidence primarily associates DEHP with generalized vascular toxicity and broad pathway disturbances (Hou et al., 2019; Zhou et al., 2022; Chu et al., 2021; Posnack, 2014). There is a lack of specific molecular evidence delineating how DEHP exposure directly engages with and dysregulates the precise gene regulatory networks that drive PAH pathogenesis. Crucially, it remains unclear whether DEHP’s effects are confined to exacerbating known inflammatory/oxidative pathways or extend to directly perturbing a distinct set of core regulatory genes specific to PAH development.

To address this gap, the present study employs an integrated systems toxicology approach. We move beyond correlative associations by combining network toxicology—to predict direct interactions between DEHP and potential protein targets—with machine learning-based bioinformatics analysis of multi-omics data from PAH. This innovative methodology is designed to bridge the disconnect between chemical exposure prediction and disease-specific driver identification. Our primary objective is to identify and prioritize a set of high-confidence core genes that are both putative direct targets of DEHP and central regulators in PAH, thereby constructing a novel molecular hypothesis for DEHP-induced PAH. This work aims to provide new, mechanism-oriented perspectives for understanding the environmental etiology of PAH and identifying potential targets for risk assessment and precision prevention.

## 2 Materials and methods

### 2.1 Acquisition of disease-related transcriptomic data

**FIGURE 1:**
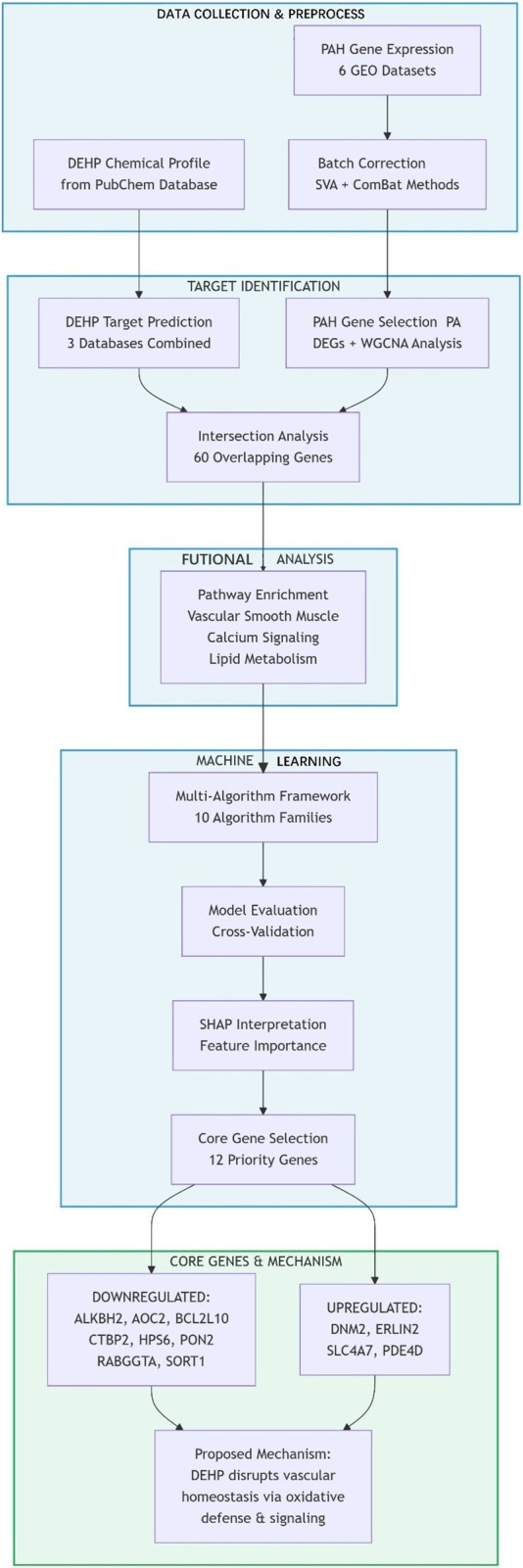
Flow-chart of datasets analysis in this paper.

Six microarray transcriptomic datasets related to PAH (GSE24988, GSE48149, GSE113439, GSE22356, GSE33463, and GSE73674) were curated from the NCBI GEO database. Datasets GSE24988, GSE48149, and GSE113439 were designated as the discovery cohort, while GSE22356, GSE33463, and GSE73674 constituted the independent validation cohort. To ensure data comparability, a multi-stage normalization pipeline was implemented to mitigate batch effects. First, Surrogate Variable Analysis (SVA) using the ‘sva’ R package was applied to the discovery cohort to model and adjust for latent confounding factors. Subsequently, the ComBat algorithm, based on a parametric empirical Bayes framework, was used to harmonize residual batch variations across all datasets. The effectiveness of normalization was confirmed by principal component analysis (PCA), which showed improved clustering of samples from different batches in a reduced-dimensional space post-correction. The complete analytical workflow is schematized in Figure 1.

### 2.2 Acquisition of chemical properties and predicted targets of DEHP

The chemical profile of DEHP as systematically characterized. Its canonical Simplified Molecular-Input Line-Entry System (SMILES) notation (CCCCC(CC)COC(=O)C1 = CC = CC = C1C(=O)OCC(CC)CCCC) and associated properties were retrieved from the PubChem database. A tripartite strategy was employed to predict potential protein targets of DEHP within the *Homo sapiens* proteome: (1) ligand-receptor interaction profiling using the ChEMBL database; (2) chemical genomics-based prediction via SwissTargetPrediction; and (3) 3D pharmacophore matching using the PharmMapper server.

### 2.3 Differential gene expression analysis

Differential expression analysis was performed on the normalized transcriptomic data using the ‘limma’ R package. Genes with an adjusted p-value (FDR) < 0.05 and an absolute log2 fold-change (|log2FC|) > 0.585 (corresponding to a 1.5-fold change) were defined as differentially expressed genes (DEGs). Results were visualized using the ‘ggplot2’ package.

### 2.4 Weighted gene Co-expression network analysis (WGCNA)

A scale-free co-expression network was constructed using the ‘WGCNA’ R package. After removing outlier samples via hierarchical clustering, an optimal soft-thresholding power was determined to achieve a scale-free topology fit index (R^2^) > 0.85. A topological overlap matrix (TOM) was then generated, and genes were clustered into modules using dynamic tree cutting with a minimum module size of 50. Modules with high similarity were merged at a cut height of 2. Module-trait associations were assessed by correlating module eigengenes with the PAH phenotype, retaining modules with a Pearson’s |R| > 0.5 and p < 0.05. Genes with high intramodular connectivity (kME >0.8) within significant modules were identified as hub genes.

### 2.5 Identification of DEHP-Associated PAH targets

To identify core targets linking DEHP exposure to PAH, an intersection analysis was performed between three gene sets: (1) PAH-related DEGs, (2) WGCNA-derived hub genes from phenotype-associated modules, and (3) computationally predicted targets of DEHP. The overlapping genes were considered potential core mediators and were visualized using Venn diagrams.

### 2.6 Functional enrichment analysis

Biological interpretation of the core target genes was conducted through functional enrichment analysis using the ‘clusterProfiler’ R package. Gene Ontology (GO) terms and Kyoto Encyclopedia of Genes and Genomes (KEGG) pathways significantly associated with the gene set (p < 0.05) were identified to elucidate the potential mechanistic roles of DEHP in PAH pathogenesis.

### 2.7 Machine learning-based screening for core diagnostic genes

A comprehensive machine learning framework was established to identify robust DEHP-associated diagnostic markers for PAH. Guided by the “no free lunch” theorem, we employed a systematic exploration strategy across 10 distinct algorithm families—including regularized linear models (Lasso, Ridge, Elastic Net), tree-based ensembles (Random Forest, XGBoost, GBM), gradient boosting (glmBoost), support vector machines (SVM), and probabilistic methods (LDA, Naive Bayes), alongside parametric selection (Stepglm). This diversity ensured a wide exploration of the hypothesis space, encompassing linear, non-linear, and interactive effects without prior bias. For each algorithm, a rigorous hyperparameter tuning grid was implemented via 5-fold cross-validation on stratified data splits, resulting in the training and evaluation of 128 distinct model instances to fairly optimize each learning approach.

Model performance was primarily evaluated using the area under the receiver operating characteristic curve (AUC), with accuracy and F1-score as secondary metrics. To construct a final, robust predictor, we implemented a multi-stage selection process: 1) High-performing models (AUC >0.9) were first selected based on cross-validated performance and calibration. 2) From this high-confidence set, predictive features (genes) were integrated using a consensus approach, ranking genes by their selection frequency across the constituent models. This strategy prioritizes features that are consistently important to diverse, well-performing algorithms, enhancing biological reliability and model stability. The final ensemble leverages the complementary strengths of the top-performing models, balancing predictive power with interpretability. Expression patterns of the identified core consensus genes were visualized in a heatmap using the “pheatmap” package.

### 2.8 Model interpretation via SHAP analysis

To enhance the interpretability of the machine learning models and quantify the contribution of individual genes to the predictions, the SHAP (SHapley Additive exPlanations) algorithm was employed. This method assigns a SHAP value to each feature (gene) for every prediction, providing a consistent and locally accurate measure of its influence on the model output, thereby overcoming the “black-box” limitation.

## 3 Results

### 3.1 Identification of potential target proteins of DEHP

**FIGURE 2:**
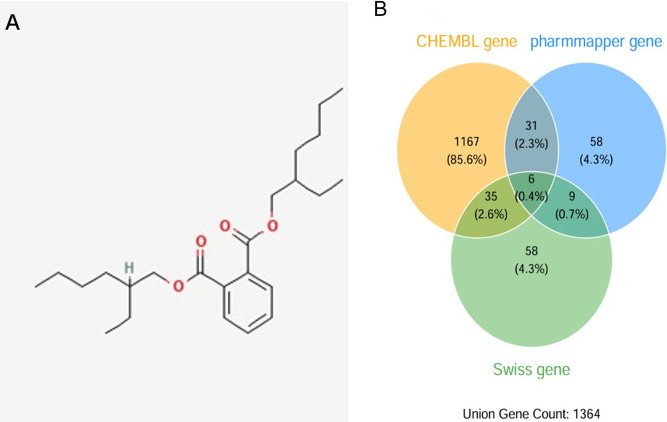
Identification of DEHP target proteins. **(A)** Chemical structure of DEHP. **(B)** Target prediction using CHEMBI, PharmMapper, and SwissTargetPrediction.

The two-dimensional molecular structure of DEHP was acquired from the PubChem database (Figure 2A). To comprehensively map its potential biological interactions, target prediction was performed by integrating results from three independent platforms: ChEMBL (for known bioactive molecule profiles), SwissTargetPrediction (for ligand-based similarity prediction), and PharmMapper (for reverse pharmacophore matching). Following consolidation and removal of duplicates, this integrated approach yielded a non-redundant set of 1,364 unique human proteins as potential targets of DEHP (Figure 2B).

### 3.2 Identification of PAH-Related differential genes and Co-Expression modules

**FIGURE 3:**
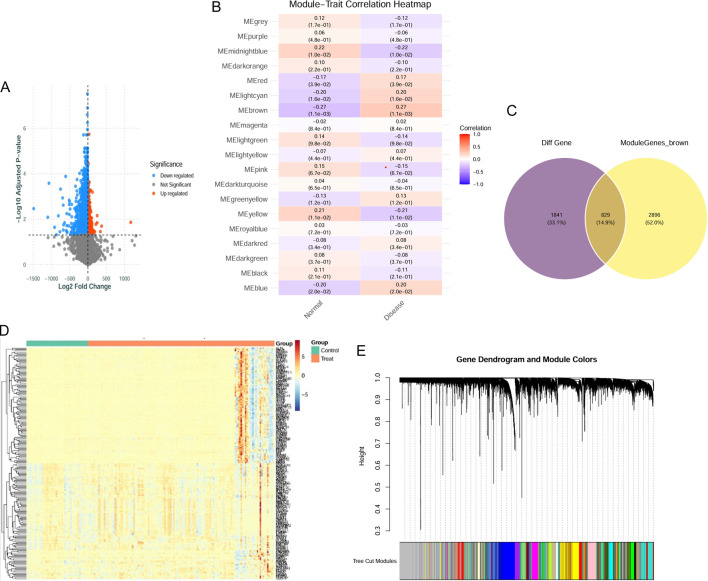
Identification of PAH-related target genes. **(A)** Volcano plot shows DEGs based on logFC and significance. Red dots are upregulated, green dots are downregulated, and grey dots are non-significant. **(B)** Module-trait relationships heatmap shows correlations between WGCNA-identified modules and sample traits (Control vs. Treatment). Values in boxes indicate correlation coefficients and p-values. **(C)** Venn diagram shows DEGs (purple) and WGCNA modules (yellow), with brown indicating common genes from both methods. **(D)** Heatmap shows DEG expression patterns across samples. Red indicates upregulation, blue indicates downregulation. **(E)** Gene dendrogram from WGCNA shows hierarchical clustering based on co-expression. Module colors in the lower panel represent different gene modules.

The gene expression matrices from datasets GSE24988, GSE48149, and GSE113439 were merged and subjected to a rigorous normalization pipeline to correct for technical batch effects. PCA confirmed the efficacy of this process, showing tighter and more distinct sample clustering post-normalization. Differential expression analysis between PAH and control samples identified 2,669 significantly dysregulated genes, visualized via volcano plots and heatmaps (Figures 3A,D). In parallel, a WGCNA was conducted. Network topology analysis determined a soft-thresholding power (β) of 5 as optimal to approximate a scale-free network (scale-free R^2^ ≥ 0.8). Hierarchical clustering of the resulting TOM delineated 19 distinct co-expression modules, each assigned a unique color (Figure 3E). Correlation analysis between module eigengenes and the PAH phenotype identified several modules with significant associations (P < 0.05, Figure 3B). The union of genes from the differential expression analysis and the significant WGCNA modules, after deduplication, provided a consolidated list of 829 PAH-associated genes for downstream analysis (Figure 3C).

### 3.3 Identification and functional enrichment of DEHP-Associated targets in PAH

**FIGURE 4:**
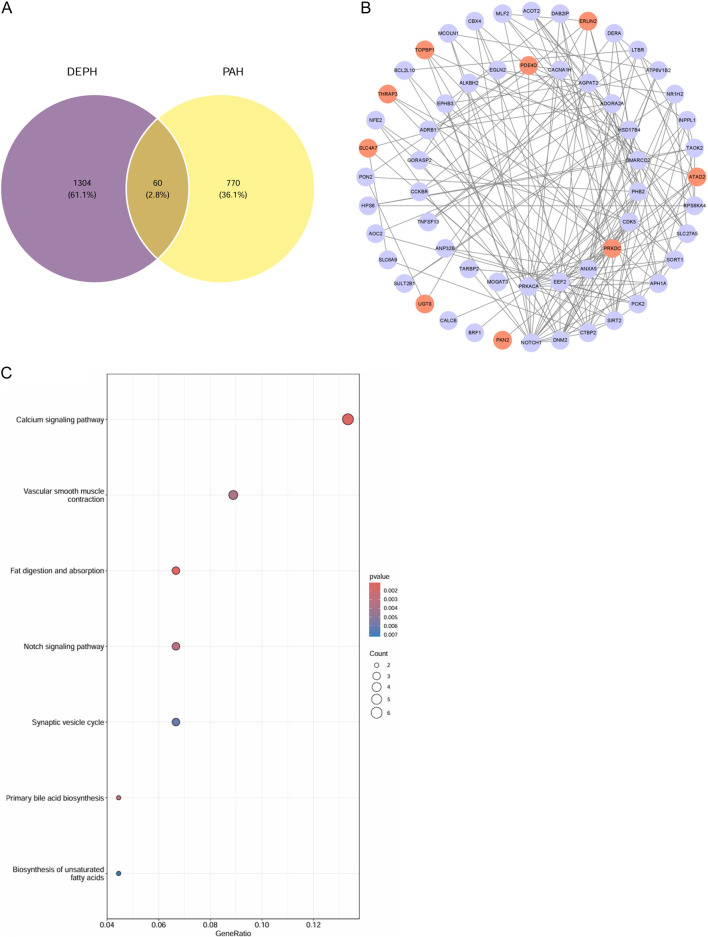
Identification of DEHP-associated disease targets in PAH. **(A)** Venn diagram compares genes linked to DEHP exposure (purple) and PAH (yellow), with 60 overlapping genes (2.8%). **(B)** PPI network visualizes interactions among overlapping genes. Red nodes = upregulated, blue nodes = downregulated, edges = predicted interactions. **(C)** KEGG analysis shows enriched pathways for overlapping genes. X-axis = gene ratio, dot size = gene count, color gradient = adjusted p-value (red = higher significance).

Venn analysis was employed to intersect the 1,364 predicted DEHP targets with the 829 PAH-associated genes, revealing 60 overlapping genes as high-confidence candidates mediating DEHP-induced PAH (Figures 4A,B). To elucidate their collective biological role, functional enrichment analysis was performed. GO terms significantly enriched among these 60 genes included ‘glycerolipid metabolic process’ and ‘fatty acid metabolic process’. KEGG pathway analysis further highlighted their involvement in critical pathways such as ‘Vascular smooth muscle contraction’ and the ‘Calcium signaling pathway’ (Figure 4C). These results implicate the dysregulation of lipid metabolism and calcium-mediated vascular tone regulation in the pathogenesis of DEHP-related PAH.

### 3.4 Machine learning-based prioritization and interpretation of core genes

**FIGURE 5:**
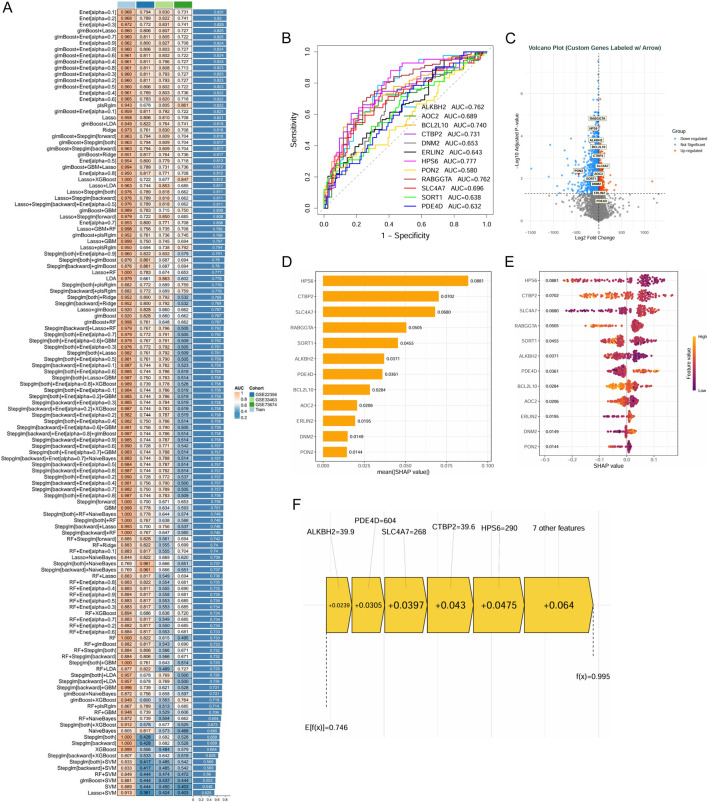
Identification of core genes in DEHP-induced PAH. **(A)** Model performance comparison: heatmap shows AUC values for various models across cohorts. Left column = models, right column = AUC (higher = better). Colors indicate cohort sources. **(B)** ROC Curves: ROC curves for key genes (ALKBH2,AOC2,BCL2L10,CTBP2, DNM2, ERLIN2, HPS6,PON2,RABGGTA,SLC4A7,SORT1,PDE4D). X-axis = false positive rate, Y-axis = sensitivity. AUC indicates predictive performance. **(C)** Volcano plot: volcano plot shows DEGs. X-axis = logFC, Y-axis = -log10(P-value). Red = upregulated, green = downregulated, with key genes labeled. **(D)** Feature importance ranking: a bar graph ranks top genes by feature importance. Larger bars = greater contribution to the model. **(E)** Violin plot: violin plot shows gene expression distributions across conditions. Width = data density, colors = expression levels. **(F)** SHAP Summary Plot: SHAP summary plot shows gene contributions to predictions. Negative SHAP = lowering effect, positive = increasing effect.

The 60 candidate genes were subsequently refined through an advanced machine learning framework. Following the construction and evaluation of 128 predictive models, an ensemble model integrating the glmBoost and Stepglm algorithms was selected based on its superior and consistent diagnostic performance in internal cross-validation (Figure 5A). This process prioritized twelve core diagnostic genes: ALKBH2, AOC2, BCL2L10, CTBP2, DNM2, ERLIN2, HPS6, PON2, RABGGTA, SLC4A7, SORT1, and PDE4D. The strong discriminative power of this gene set was confirmed by ROC curve analysis (Figure 5B), and their differential expression profiles in PAH are visually summarized in a volcano plot (Figure 5C).

To interpret the ensemble model and quantify the contribution of each feature, SHAP (SHapley Additive exPlanations) analysis was employed. This identified HPS6 (mean |SHAP| = 0.0881) and CTBP2 (mean |SHAP| = 0.0702) as the most influential positive predictors, where their elevated expression levels contributed to a higher model output (i.e., increased PAH risk score) (Figures 5D,E). Individual prediction analysis further illustrated this driver effect: in a representative high-risk prediction (f(x) = 0.995), the specific expression values of HPS6 and CTBP2 provided the largest positive shifts (ΔSHAP = +0.0475 and +0.043, respectively), pushing the prediction well above the baseline expected value (E [f(x)] = 0.746) (Figure 5F).

## 4 Discussion

This study employed an integrative computational approach, combining network toxicology with machine learning-based bioinformatics, to investigate the molecular interface between the ubiquitous plasticizer DEHP and PAH. We identified a cohesive set of 12 core genes whose expression is predicted to be significantly dysregulated by DEHP and which are concurrently central to PAH-associated networks. This signature, comprising ALKBH2, AOC2, BCL2L10, CTBP2, DNM2, ERLIN2, HPS6, PON2, RABGGTA, SLC4A7, SORT1, and PDE4D, provides a novel, high-priority hypothesis that moves beyond generic associations of DEHP with oxidative stress. Instead, it posits that DEHP may instigate PAH-relevant pathology by coordinately disrupting specific biological processes crucial for vascular homeostasis, including cellular repair, vesicular trafficking, and second-messenger signaling.

Several key functional clusters emerge. First, genes involved in cellular stress response and repair are suppressed. The downregulation of PON2, a known protector against oxidative stress in endothelial cells (Altenhöfer et al., 2010), and ALKBH2, a DNA alkylation repair enzyme (Johannessen et al., 2013), suggests a dual impairment of defense mechanisms. This could lower the threshold for persistent oxidative and genotoxic damage in pulmonary vascular cells, a permissive state for dysfunction. Second, genes regulating vesicular transport and protein sorting are perturbed. SORT1 (downregulated) influences lipid metabolism and receptor trafficking, including potential modulation of growth factor receptors (Coutinho et al., 2013; Carlo, 2013), while DNM2 (upregulated) is essential for clathrin-mediated endocytosis. Their coordinated dysregulation could disrupt precise spatiotemporal signaling, potentially affecting pathways like TGF-β/BMP through altered receptor dynamics (Ferguson and De Camilli, 2012; Mcmahon and Boucrot, 2011). Third, the upregulation of signaling modulators like PDE4D points to a direct impact on vascular tone and cell proliferation. PDE4D hydrolyzes cAMP, a key vasodilatory and anti-proliferative second messenger in the pulmonary vasculature (Houslay and ADAMS, 2003; Lehnart et al., 2005; Pullamsetti et al., 2013). Its overexpression would diminish cAMP signaling, promoting vasoconstriction and smooth muscle cell growth—hallmarks of PAH.

This multi-target model suggests DEHP does not merely induce a generalized inflammatory/oxidative state but may orchestrate a specific pathological program by simultaneously weakening cellular repair (ALKBH2, PON2), distorting intracellular communication (SORT1, DNM2), and shifting signaling balance toward constriction/proliferation (PDE4D, SLC4A7). This program likely interacts with and exacerbates classical PAH pathways. For instance, the downregulation of the transcriptional corepressor CTBP2 could dysregulate networks governing cell proliferation and fate, potentially synergizing with compromised BMPR2 signaling [Ref to CTBP2 function in other contexts] (Chinnadurai, 2002).

It is noteworthy that among the 12 predicted core targets of DEHP, only two (SLC4A7, PDE4D) were also differentially expressed in an independent clinical PAH transcriptomic dataset (GSE113439). Furthermore, there was no overlap with classical heritable PAH genes (such as BMPR2, ACVRL1, ENG, SMAD9, CAV1, KCNK3, TBX4, and EIF2AK4) (Humbert et al., 2022). This suggests that DEHP may promote pathological progression through alternative “non-classical” targets, ultimately converging on the terminal pathway of vascular remodeling, profoundly revealing the complex nature of environment-driven disease.

By combining DEHP target prediction (network toxicology) with disease-specific driver identification (machine learning based on pulmonary hypertension omics data), we more directly connected chemical exposure to disease pathogenesis than with purely correlative approaches (Jeong and Choi, 2022). This research workflow is particularly suitable for studying complex diseases such as pulmonary hypertension—where environmental triggers may disrupt their nonlinear interconnected networks (Escher et al., 2017; Schadt, 2009).

The findings must be viewed within the context of mixed environmental exposures. DEHP often co-occurs with other PAH-associated pollutants like particulate matter and PAHs (Zhou et al., 2022; García-Garcinuño et al., 2024). These agents share upstream effector mechanisms like oxidative stress induction. Therefore, the identified core gene network may represent a DEHP-preferential but not exclusive pathway that could be amplified in co-exposure scenarios, leading to synergistic vascular injury (Han et al., 2023; Deng et al., 2019; Quirós-Alcalá et al., 2023; Sarmiento and Majestic, 2023). This underscores the importance of investigating pollutant mixtures to fully understand environmental PAH risk.

The main limitation of this study lies in its reliance solely on computer simulation analysis and machine learning predictions, lacking wet lab experimental validation. The specific binding affinity of DEHP with these targets, its direct regulatory role in pulmonary vascular cells, and the resulting phenotypic changes still require further validation through experimental methods such as molecular docking, cell knockout/overexpression experiments, Western blotting, and *in vivo* models.

## 5 Conclusion

These findings demonstrate that DEHP promotes PAH pathogenesis by modulating specific genes and associated pathways. The twelve core genes identified through machine learning are proposed as key regulators in this process, providing crucial insights for future mechanistic investigation into DEHP-induced PAH.

## Data Availability

The original contributions presented in the study are included in the article/Supplementary Material, further inquiries can be directed to the corresponding author.
